# Comparison of the serum brain-derived neurotrophic factor (BDNF) between fibromyalgia and nociceptive pain groups; and effect of duloxetine on the BDNF level

**DOI:** 10.1186/s12891-022-05369-8

**Published:** 2022-05-02

**Authors:** Ali Bidari, Banafsheh Ghavidel-Parsa, Faeze Gharibpoor

**Affiliations:** 1 Department of Rheumatology, Iran University of Medical Sciences, Tehran, Iran; 2 Rheumatology Research Center, Razi Hospital, School of Medicine, Guilan University of Medical Science, Rasht, Iran; 3 Student Research Committee, Deputy of Research and Technology, Faculty of Medicine, Guilan University of Medical Sciences, Namjoo St, Rasht, Guilan 41446-66949 Iran

**Keywords:** Fibromyalgia, Nociceptive pain, BDNF, Duloxetine, Nociplastic pain

## Abstract

**Background:**

The primary objective was to compare the serum brain-derived neurotrophic factor (BDNF) level in the patients with two types of pain: fibromyalgia (FM) and non-FM nociceptive pain (non-FM NP). The secondary objective was to investigate the effect of duloxetine on serum BDNF in FM patients and assess the direction of BDNF changes’ relation to clinical parameters’ alterations.

**Methods:** This is a study on 73 patients (50 FM and 23 non-FM chronic non-inflammatory pain patients). Serum BDNF was first compared between both groups. Patients with FM, then prospectively, underwent standardized FM treatment with duloxetine maximized to 60 mg/day. The Revised Fibromyalgia Impact Questionnaire (FIQR), Short-Form Health Survey (SF-12), pain visualized analog scale (pain VAS), Beck Depression Inventory-II (BDI-II), polysymptomatic distress scale (PSD) and serum BDNF were measured and compared at baseline and 4 weeks after treatment in FM group.

**Results:**

The mean of adjusted BDNF level in the FM group had no significant difference than the non-FM NP group ((5293.5 ± 2676.3 vs. 6136.3 ± 4037.6; *P* value = 0.77). Using linear mixed model, we showed that duloxetine reduced BDNF level significantly in FM patients, even after adjusting for depression, pain and severity of the disease (*P* < 0.01). The FIQR, BDI-II, PSD, and pain VAS improved significantly after duloxetine treatment.

**Conclusions:**

Non-significant BDNF level difference between FM and non-FM nociceptive pain suggested that peripheral BDNF is not a pathophysiological feature of FM. The decreased BDNF level parallel with improvement of PSD/pain scores after duloxetine treatment indicates BDNF alteration in the pain modulation process, regardless of cause and effect.

## Background

Fibromyalgia (FM) is a chronic pain disorder consisting of musculoskeletal pain and hyperalgesia, commonly accompanied by sleep disturbance, fatigue, anxiety, depression, gastrointestinal symptoms and headache [1]. Recently, FM has been classified into the nociplastic pain category which incorporate the pain conditions with altered nociception without clear evidence of peripheral or central somatosensory damage [2]. Many efforts have been devoted to identifying biomarkers explaining the nociplastic mechanisms and increased global sensory hypersensitivity in the FM. These investigations have led to the growth factors as an important player in neuronal survival and plasticity [3–7].

Brain-derived neurotrophic factor (BDNF), as the most abundant and widely distributed neurotrophin in the central and peripheral nervous system, has been known to be involved in axonal growth, synaptic plasticity, and neuronal repair [7, 8]. Recent evidence has shown that BDNF has a strong role in cognitive functions, notably in memory acquisition and consolidation [9]. Despite the numerous data investigating the link between BDNF and chronic pain, the conflicting results have led to fuzzy and complexity of this relationship. The circulatory BDNF level, which mainly originates from various regions of the central nervous system (CNS), has been suggested to be altered in chronic pain conditions [10, 11]. Accumulating data of circulating and cerebrospinal fluid (CSF) levels of BDNF in the FM patients shows contradictory results [12–16]. Although a higher level of BDNF in the patients with FM rather than healthy subjects was reported in the former studies [12–15], some recent studies reported a normal level of BDNF in the FM [16]. Furthermore, it remains to be clear whether the direction of BDNF changes in the nociceptive pain type differs from the nociplastic FM pain. From the clinical perspective of pain type, the nociceptive pain is pain originating from actual damage to non-neural tissue (such as mechanical or inflammatory musculoskeletal pain). It proportionates with tissue mechanical or anatomical damage and is not prominently associated with sensory hypersensitivity and central sensitization. The prototype example of nociceptive pain is the pain originating from joints with mechanical and inflammatory damages [2]. It could be hypothesized that BDNF, proposed as the involved neurotrophic factor in central sensitization, potentially acts differently and may not be significantly involved in the nociceptive pain process.

On the other hand, the strong clinical link between pain and depression adds more complexity to the relationship between BDNF and FM nociplastic pain. Pain and depression frequently coexist in patients and have mutual effects [17]. Given many studies which found lower BDNF levels in the serum and CSF of the patients with depression [18–20], this question is raised: how does BDNF level change in the patients with chronic pain, including FM with or without depression? Certainly, given the heterogeneous nature of the FM and depression, it seems hard to formulize the relation of the BDNF with pain and depression.

In order to elucidate the complex relationship between BDNF, pain, and depression in the nociplastic FM and chronic nociceptive pain, it seems reasonable to explore the effect of approved medications for FM and depression on the BDNF level in the painful conditions and also to assess the relationships of BDNF changes with the clinical indices’ improvement. Numerous studies have investigated the effect of antidepressants on the BDNF level of patients with depression. Although the majority claim the increased level of BDNF after the treatment, some have elucidated different results [19–22]. Similar data on FM patients with/without depression treated with a low analgesic dose of antidepressants has revealed inconsistent results [13–15]. To the best of our knowledge, no prospective study has investigated the effect of antidepressant medications on the serum level of BDNF in FM patients with and without depression.

Considering the mentioned debate, we sought to compare the serum BDNF level in the patients with two different types of pain: nociplastic FM pain and nociceptive pain (non-inflammatory chronic rheumatic pain). We also investigated the effect of the FM-approved antidepressant, serotonin–noradrenaline reuptake inhibitor (SNRI), duloxetine, on the serum BDNF in patients with FM and also assessed the direction of serum BDNF changes to the clinical symptom severity.

## Materials and methods

### Design and setting

This prospective study was conducted in the academic outpatient rheumatology clinic, Razi hospital affiliated with Guilan University of Medical Sciences (GUMS), from May 2019 through October 2019. The study protocol was approved by the ethics committee of Iran University of Medical Sciences (IUMS) in accordance with the World Medical Association’s code of ethics (Declaration of Helsinki, revised in Brazil 2013).

### Participants

53 FM patients and 23 patients with non-FM chronic nociceptive pain disorders (non-FM NP) were consecutively included. The diagnosis of all participants was made by two rheumatologists (A.B and B.GH), who were experts in diagnosing and managing FM and chronic pain disorders. Patients with a new diagnosis of FM based on the 2016 American College of Rheumatology (ACR) were eligible in the FM group. Patients in the non-FM chronic pain group were subjects with a chronic painful non-inflammatory condition such as osteoarthritis, tendinitis (such as lateral or medial epicondylitis, adhesive capsulitis, etc.) and had no concurrent diagnosis of FM at the time of enrollment. In order to avoiding the confounding factors, only female patients were selected in both FM and non-FM groups. Systemic inflammatory rheumatic diseases were not recruited in the non-FM NP group because several reports imply inflammation’s influence on the BDNF level and function [23]. Inclusion criteria were women with ages ranging between 18 to 65 years old, no history of antidepressant consumption within 12 weeks, not using muscle relaxants, steroids, opioids, analgesics and benzodiazepines within 1 week and monoamine oxidase inhibitors within 2 weeks prior to the study.

Participants were excluded if they were pregnant or breastfeeding. Patients with major comorbidity, including systemic inflammatory rheumatic disease, malignancies, multiple major surgeries or trauma injuries, neurological or psychiatric disorders except for depression/anxiety, chronic liver or renal diseases and hypersensitivity to duloxetine were excluded. Written informed consent was obtained from all patients, and they were informed that their level of care wouldn’t be affected if they quit the study.

### Sample size

The sample size was calculated using G*Power 3.1. To calculate the effect size, we used the study result by Laske et al., which showed an effect size of 0.96 with 80% power [15]. Reducing the effect size of Cohen’s d to 0.8 with 80% power (alpha: 0.05, two-tailed), G*power suggested the total sample size of 58 participants (allocation ratio of 2:1). The final sample size with 20% dropout was 24 control and 48 FM patients.

### Questionnaires and interventions

Demographic data (including age, educational level, marital status and work status) was obtained from all participants. The contact telephone number was also received to remind patients of their appointments. All the patients were asked to fill out the Revised Fibromyalgia Impact Questionnaire (FIQR), Short-Form Health Survey (SF-12), pain visualized analog scale (pain VAS), Beck Depression Inventory-II (BDI-II), polysymptomatic distress scale (PSD) at the initial visit. An experienced medical assistant offered help if patients did not understand the meaning of the questions.

The FIQR is a 21-items questionnaire with an 11-point numeric rating scale for each question which assesses clinical symptom severity and disease impact in patients with FM [24]. The total score of the FIQR ranges between 0 and 100, with a higher score indicating worse disease impact. The SF-12 questionnaire evaluates the health status, including the mental and physical health domains, with eight scales. Scores range from “0 to 100” where “0” indicates the worst condition and “100” indicates the best possible condition [25]. The BDI-II is composed of 21 questions, each scored 0–3 (sum = 0–63), with higher scores indicating more severe depressive symptoms over the past 2 weeks prior to the assessment. The scores above 17 were considered as the depression condition [26]. The PSD, also known as the FM severity score, indicates the fibromyalgianess regardless of FM diagnosis and could be used in other painful conditions for measuring the magnitude and severity of FM symptoms. It is the sum of the widespread pain index (WPI) and symptom severity scale (SSS) [27].

FM patients were assigned to receive Cymbalta® (Istanbul, Turkey) (duloxetine) for 4 weeks. The regimen for duloxetine started with 30 mg (mg) per day for the first week and was titrated to 60 mg per day for the next 3 weeks if no adverse effect was reported. The FM group was asked to complete the mentioned questionnaires again 4 weeks later to assess the changes in the scores after treatment with duloxetine. Of the FM group, 50 patients completed the 4 weeks of the treatment.

### Measurement of serum BDNF level

Peripheral venous blood samples (10 ml) were gathered from all participants into tubes with no anticoagulation. Patients were on 8 hours of fasting, and the samples were taken at 8 to 11 am. The time gap between sample collection and centrifuge was kept under 30 minutes to reduce platelete-derived BDNF. The samples were centrifuged at 2000 rounds per min for 10 minutes and was stored at − 80 degrees Celsius until analysis. The analysis of all samples were performed after 4 weeks from the day that the last participant was recruited [28]. Serum levels of BDNF were measured using enzyme immunoassay—the commercially available Human BDNF immunoassay system kit (ABCAM, Cambridge, UK), according to the manufacure’s protocole. To reduce inter-assay variability, each plate had a standard curve from the same solution provided by the ELISA kit. The final samples’ concentration was calculated from a single standard curve. This standard curve was the average of all standard curves obtained from each plate. In addition, each individual’s levels of BDNF (before and after treatment) were evaluated in a same assay. All assays were also performed by one ELISA-experienced laboratory technician on the same day. All sample and standard curves were measured in a duplicate manner and statistical analysis was performed on the mean values. The detection limit was 2.4 pg/ml. The observed intra- and inter-assay coefficient variances were less than 10%. All laboratory analysis and blood sampling were conducted in a private laboratory, accredited by health ministry (JAM pathobiology and genetics laboratory, Rasht, Iran).

### Statistical analysis

Descriptive statistics were used to calculate the mean ± standard deviation for continuous variables and frequency for categorical variables. To check the normality of variables, we used the Kolmogorov-Smirnov test. Skewed variable distributions were transformed by taking the cube root. Levene’s test was used to check if samples had equal variances.

Paired t-test and Wilcoxon signed-rank test were used to compare two related groups for normally and non-normally distributed data, respectively. To compare two unrelated groups, we used an independent t-test for normally distributed data and Mann-Whitney U for non-normally distributed data. For comparing the relationship between two continuous data, Pearson or Spearman was used for normally and non-normally distributed data, respectively. A univariate linear model, adjusted for FIQR, BDI-II, and VAS, was used to compare the serum BDNF level between case and control groups.

A linear mixed model was developed to compare the serum level of BDNF before and after treatment with duloxetine, adjusted for FIQR, BDI-II, and pain VAS. The equation of LMM consists of two levels:

The equation in level 1

$${y}_{ij}={b}_{0i}+{b}_{1i} tim{e}_{ij}+{e}_{ij}$$

The equation in level 2.

$${b}_{0i}={\beta}_{00}+{\beta}_{01} FIQR+{\beta}_{02} VAS+{\beta}_{03} BDI-II+{\upsilon}_{0i}$$

*b*_1*i*_ = *β*_10._

Note: y_ij_ denotes individual is serum BDNF level at time j where j = 1,2 represents measurement times. In level 1, only time-varying covariate (time) is included and it indicates each individual’s growth trajectory of outcome measure b_1i_. Each individual’s response at baseline (b_0i_) is allowed to differ by releasing its random effect in level 2 (*υ*_0*i*_). In level 2, the regression coefficient (*β*_01_, *β*_02_, *β*_03_) represents that each participants’ initial status (intercept) will be associated with their covariates. The regression coefficient (*β*_10_) indicates the time effect. Several variance-covariance structures were examined for obtaining the best fit. To make this selection, we used Bayesian information criteria (BIC), which would show the least value. Autoregressive of Order 1 Ar (1) was chosen based on this criterion. Parameters were estimated by the method of restricted maximum likelihood (REML).

Statistical calculations were performed using the IBM SPSS Statistics for Windows, version 24 (IBM Corp., Armonk, N.Y., USA), and statistical significance was evaluated at the level of 0.05.

## Results

### Patient characteristic

**Fig. 1:**
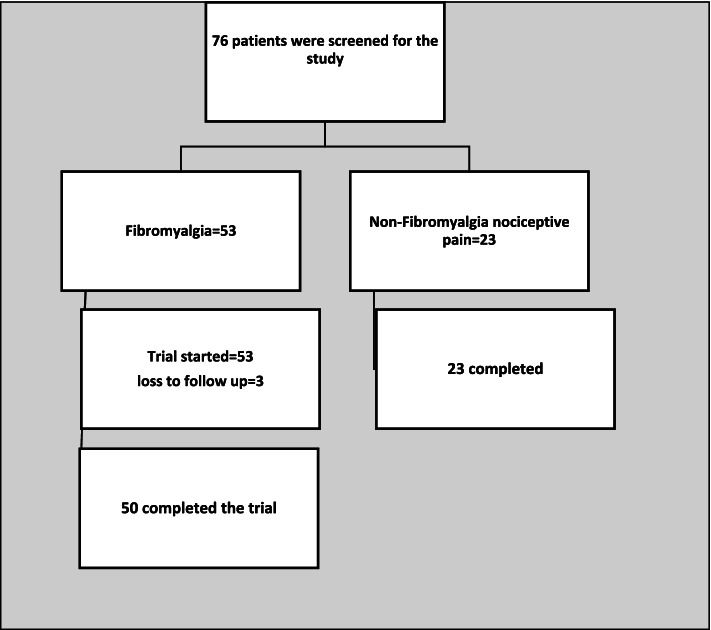
Flow diagram of participants

Of 53 patients with FM included in the FM group, 50 patients completed the 4 weeks course of treatment with duloxetine; three patients left the study before the follow-up visit (Fig. 1). Twenty-three patients with non-inflammatory chronic pain disorders were included in the non-FM NP group. There was no significant difference in terms of age, years of education and marital status between the two groups (*p* value > 0.05). As expected, the FM patients had significantly higher mean scores than the non-FM NP patients in the FIQR scores (53.6 ± 19.0 vs. 20.6 ± 13.6; *p* value< 0.01), the BDI-II scores (17.0 ± 10.0 vs. 6.4 ± 6.1; *p* value< 0.01), and the PSD scores (14.8 ± 4.6 vs. 4.9 ± 2.6; *p* value< 0.01). The health status was also considerably worse in the FM patients (45.7 ± 15.4 vs. 53.1 ± 9.5; *p* value = 0.01) and (35.3 ± 11.0 vs. 44.7 ± 11.8; *p* value< 0.01) for mental and physical components, respectively. (Table 1).

**Table 1:** Baseline patient characteristics

Variable	FM (*n* = 50)	Non-FM NP (*n* = 23)	*P* value
Age, years	46.3 (9.4)	47.6 (11.8)	0.58
Education, years			
0–8	28% (14)	26% (6)	0.50
8–12	42% (21)	48% (11)	
> 12	30% (15)	26% (6)	
Marital Status			
Single	20% (10)	13% (3)	0.70
Married	80% (40)	87% (20)	
Employment Status			
Unemployed	86% (43)	87% (20)	0.44
Employed	14% (7)	13% (3)	
FIQR	53.6 (19.0)	20.6 (13.6)	< 0.01
BDI-II	17.0 (10.0)	6.4 (6.1)	< 0.01
PSD	14.8 (4.6)	4.9 (2.6)	< 0.01
Pain VAS	7.1 (2.0)	4.5 (2.7)	< 0.01
SF-12			
Mental Component	45.7 (15.4)	53.1 (9.5)	0.01
Physical Component	35.3 (11.0)	44.7 (11.8)	< 0.01
Serum BDNF^*^(pg/ml), Mean (SD)	5293.5 (2676.3)	6136.3 (4037.6)	0.77^*^

Values are mean (standard deviation), or percent (number), *adjusted for FIQR, BDI-II and VAS: FIQR: Revised Fibromyalgia Impact Questionnaire, Beck Depression Inventory-II, PSD: polysymptomatic distress scale, Pain VAS: Pain visual analog scale, 12-Item Short-Form Health Survey (SF-12). *P* value < 0.05 is statistically significant

While the mean serum BDNF level in the FM group was lower than the non-FM NP group (5293.5 ± 2676.3 vs. 6136.3 ± 4037.6; *p* value = 0.68), this difference was non-significant even after adjusting for FIQR, BDI-II, and pain VAS as covariates (*p* value = 0.77) (Table 1).

### Relationship between serum BDNF level and clinical parameters

There was no significant relationship between the serum level of BDNF and age (*p* value = 0.38). When assessing all participants, an overall trend of decrease in the level of serum BDNF with increasing the level of FIQR, BDI-II, PSD, and pain VAS was seen. This relationship was not statistically significant (*p* value > 0.05). A similar relationship was also noted in the FM group except for the pain PSD and pain VAS, which were statistically significant (*p* value < 0.05) (Table 2).

**Table 2:** Correlations between baseline serum BDNF and clinical parameters

BDNF &:	Correlation Coefficient	*P* value
Age		
All participants(*n* = 73)	0.10	0.38^¥^
FM group(*n* = 50)	0.05	0.68^¥^
FIQR		
All participants(*n* = 73)	−0.12	0.31^¥^
FM group(*n* = 50)	−0.25	0.07^¥^
BDI-II		
All participants(*n* = 73)	−0.08	0.50^¥¥^
FM group(*n* = 50)	−0.11	0.42^¥^
PSD		
All participants(*n* = 73)	−0.19	0.09^¥^
FM group(*n* = 50)	−0.31	0.02^*¥^
Pain VAS		
All participants(*n* = 73)	−0.20	0.08^¥¥^
FM group(*n* = 50)	−0.32	0.02^*¥¥^

^¥^ These values are based on Pearson analysis, ^¥¥^ These values are based on Spearman analysis, FIQR: Revised Fibromyalgia Impact Questionnaire, BDI-II: Beck Depression Inventory-II, PSD: polysymptomatic distress scale, Pain VAS: Pain visual analog scale. *P* value is statistically significant at the 0.05 level (two-tailed)

### Effect of duloxetine on the outcome values

Our results showed that one-month treatment with duloxetine significantly improved the FIQR, BDI-II, PSD, and pain VAS scores (Table 3). The mean levels of serum BDNF decreased significantly after treatment with duloxetine in the FM patients (*p* value< 0.01) (Table 3). Using a linear mixed model, we showed that duloxetine reduced serum BDNF level significantly, even after adjusting for depression, pain and severity of the disease (*p* value< 0.01) (Table 4).

**Table 3:** Comparison of serum BDNF level and clinical parameters before and after treatment with duloxetine

Value	Before treatment	After treatment	Statistic
FIQR	53.6 (19.0)	36.4 (22.9)	*P* < 0.01
BDI-II	17.0 (10.0)	13.9 (11.3)	*P* = 0.01
PSD	14.8 (4.6)	8.4 (3.3)	*P* < 0.01
Pain VAS	7.1 (2.0)	4.4 (3.0)	*P* < 0.01
Serum BDNF (pg/ml)	5293.5 (2676.3)	3608.2 (2584.6)	*P* < 0.01^*^

Values are mean (standard deviation), ^*^ This value is based on a linear mixed model adjusted for BDI-II, FIQR and pain VAS. FIQR: Revised Fibromyalgia Impact Questionnaire, BDI-II: Beck Depression Inventory-II, PSD: polysymptomatic distress scale, Pain VAS: Pain visual Analog scale. *P* value < 0.05 is statistically significant

**Table 4:** Summary table of linear mixed model for effect of duloxetine on serum BDNF level

Variable	Estimated coefficient	SE	T	*P* value
Intercept	60.38	5.20	11.60	< 0.01
Time	16.15	3.74	4.31	< 0.01
FIQR	−0.03	0.12	−0.24	0.81
BDI-II	0.19	0.22	0.88	0.38
Pain VAS	−1.16	0.98	−1.18	0.24

*SE* Standard error, *FIQR* Revised Fibromyalgia Impact Questionnaire, *BDI-II* Beck Depression Inventory-II, *Pain VAS* Pain Visual Analogue Scale, *P* value < 0.05 is statistically significant

## Discussion

This study showed that the mean adjusted serum level of BDNF l for disease severity and depression in the FM group did not significantly differ from the non-FM NP group. Surprisingly, duloxetine decreased serum BDNF level significantly in the FM patients even after adjusting for the disease severity, depression, and pain level. Decreasing serum BDNF after treatment with duloxetine was associated with the improvement of the disease severity, depression, and pain level.

To the best of our knowledge, this is the first study comparing serum BDNF levels between different types of pain: nociplastic FM pain with prominent neuroplasticity and pain centralization, nociceptive pain with prominent peripheral explanation for pain and low pain centralization. We found that the FM group had a lower serum level of BDNF rather than the non-FM NP group. Even so, this difference turned out to be non-significant when adjusted for depression, disease severity, and pain as the main cofounders. Contrary to the previous studies which compared BDNF level in the FM patients with the healthy subjects without pain, we chose the non-FM group from patients with NP to elucidate the role of serum BDNF more clearly in the different pain types. Most previous studies implied an increased peripheral and CSF level of BDNF in FM patients compared to healthy controls [12–15], and recently some studies have claimed normal plasma levels of BDNF compared with healthy subjects [16]. Although existing data implies that BDNF changes differently in different locations (central or peripheral) under pain conditions [3, 7, 17], peripheral BDNF has been suggested to be an indicator of peripheral sensitization in the chronic pain population [7, 29]. Despite many studies which support the pro-nociceptive role of BDNF in the pain processes in the periphery and spinal cord dorsal horn and consequently predict the increased BDNF levels in these sites, it is interesting and contrary that the depression decreased peripheral level of BDNF [17–20]. The lower mean of BDNF level in our FM group could be related to the higher depression scores in the FM patients rather than the NP group. Notably, serum level of BDNF showed a non-significant difference between two pain groups (FM and NP) after adjustment for depression, disease severity and pain intensity. It could indicate the similar levels of peripheral BDNF, independent of pain types, nociplastic or nociceptive pain. This finding is congruent with the Baumeister et al.’s study [16] that found the normal plasma level of BDNF in the FM patients and suggested that BDNF is not a pathophysiological feature of FM. It may weaken the underpinnings of BDNF theory in the development of only central pain sensitization [16].

Notably, we found that the basal serum level of BDNF did not correlate significantly with symptom severity, PSD scores or fibromyalgianess, depression and pain severity regardless of the diagnosis (FM or non-FM NP). However, some small inverse correlations between BDNF and pain/PSD values were seen only in the FM patients. These findings were congruent with the Haas et al.’s study [13], which showed no correlation between the plasma level of BDNF and depression levels but contrasted with Nugraha et al.’s study [14], which found a positive correlation between serum level of BDNF and depression in the FM group. However, a significant negative correlation between depression scores and serum BDNF level was found in the patients with depression in the Shimizu et al.’s study [19]. Our results with an agreement with Shimizu et al.’s study [19] indicated inverse relationships of depression/disease severity indexes with BDNF level, although non-significant. The heterogeneous population of FM with genetic polymorphism and various neurotransmitters expression explains that why it is hard to predict correlations of various neurobiological reflections such as BDNF with clinical parameters in FM. It would be expectable that these correlations be simpler to predict in other pain types such as nociceptive pain in which the substantial confounding factors such as psychobiological or symptoms’ multiplicity do not exist.

Apart from BDNF level assessment in the different pain groups, we assessed the effect of duloxetine treatment on the BDNF level in our FM patients. This is the first study that has prospectively evaluated serum level of BDNF alteration in FM patients after treatment with an antidepressant. Surprisingly, we found that serum BDNF reduced after treatment with duloxetine in the FM patients. This effect remained even after adjusting for the disease severity, depression, and pain level. There have been a few retrospective studies on the issue in FM papulation that have suggested independence changes of the BDNF to antidepressant medications [13–15, 30]. Haas et al.’s study [13] found no difference in plasma level of BDNF between the antidepressant-naive FM group, patients receiving analgesic doses of tricyclic antidepressants and patients with antidepressants at therapeutic doses for depression. Low numbers of patients in each group in the Haas et al.’s study [13] and also in similar studies must be considered in the interpretation of their results. Notably, majority of the preexisting studies evaluated the effect of various antidepressants on BDNF level in patients with depression without pain. The prevailing results were the increased peripheral BDNF after pharmacological treatment, suggesting the reversion of down-regulation of peripheral BDNF levels in the depressed patients after a period of antidepressants [20–22]. In these studies, the applied dose of antidepressants was higher than those used in pain conditions such as FM. Furthermore, there is still controversy regarding the types, time course and dose of antidepressants, as well as pain existence on the BDNF level changes. In our study, the FM patients received a low dose or analgesic dose of duloxetine up to 60 mg for short-term (1 month). So, it will be conceivable that the results may change with a higher dose or longer duration of antidepressants. It has been known the analgesic effect of antidepressants is achieved earlier and with the lower dose of these drugs rather than antidepressant effects. Moreover, it remains to be clear how are the peripheral and central neurobiological changes after the analgesic dose of antidepressant treatment and how long is needed for these changes. From the perspective of depression treatment, the therapeutic effects of antidepressant drugs have been attributed to the increase in the proliferation of neuronal progenitor cells through mechanisms involving up-regulation of hippocampal BDNF levels [31, 32]. This process seems time-spending and probably needs a higher dose of these drugs. Given the known role of BDNF in pain modulation, especially its role as a defense mechanism for pain, we surmised that the decreased serum BDNF after treatment in the FM patients could be related to pain modulating, not the improvement in depression and related neuroplasticity, because depression improvement needs more time and higher dose of antidepressants. It might be theorized that decreased serum BDNF after therapy with low-dose antidepressants may be related to the analgesic-induced BDNF changes through the brain structures such as spinal and cortical modulating pain areas. These explanations for BDNF drop-off after treatment with duloxetine in our population need to be examined in future well-designed studies with different time points’ measurements of circulatory and central BDNF in FM patients.

It is noteworthy that one of the weak points in the data comparison of the BDNF level in different studies is the numerous factors that affect the measurement or interpretation of BDNF. Circulatory BDNF is measured in the serum or plasma. Plasma contains platelets accounting as the major peripheral reservoir of BDNF. The studies based on the serum level of BDNF typically showed a higher level than the plasma level. This discrepancy was probably due to the coagulation process prior to centrifugation in the process of obtaining serum samples. So, it seems the BDNF storage in serum or plasma is representative of the different sources. It has not been known that serum BDNF mainly originates from the platelet release or is derived from CNS with passing through the brain-blood barrier. There is still debate whether the plasma and serum level of BDNF are related together and whether the circulating levels of the BDNF reciprocate its CNS level. However, due to the difficulty in the CNS measurement of BDNF level, most studies prefer to use plasma or serum samples [10].

There are some limitations to this study. First, only female patients were recruited, and therefore the study findings cannot be generalized to men with painful conditions. Second, we evaluated our patients in a tertiary care setting; thus, they probably do not reflect the general population of FM or non-FM NP. Third, the non-FM NP was a relatively small group with 23 patients due to strict inclusion criteria of not having concurrent FM for entering the patients into this group. The tracing of any symptom connoting nociplastic features of pain such as unrefreshed sleep or sleep disorders, fatigue, migratory and non-consistent pain, various somatic symptoms etc., led to the exclusion of the patients from the non-FM NP group. So, this strict criteria for inclusion of patients into the non-FM NP led to the low numbers of patients in this group. Fourth, a relatively short time (1 month) for the second BDNF checking after duloxetine prescription limited us in the extrapolation of our results to long-term effects of antidepressant treatment. Because of the aforementioned serum BDNF changes involved in brain neuroplasticity after starting antidepressants, which may need long-term therapy, the results may be different in various time points’ sampling of serum BDNF. Finally, interpretations of all BDNF intergroup and intragroup comparisons were based on the peripheral (serum) BDNF, which might not be representative of the central nervous system BDNF changes.

## Conclusions

In conclusion, we found no significant adjusted serum level of BDNF difference between nociplastic FM and the nociceptive pain process. This finding re-emphasizes that peripheral BDNF is not a pathophysiological feature of FM and may weaken the theory of exclusive role of peripheral BDNF in nociplastic pain. FM as a heterogenic condition having both prominent pain and depression seems unpredictable to BDNF level measurements regarding different situations and treatments. The decreased serum level of BDNF after treatment with duloxetine in FM patients supports the overall role of BDNF in pain modulation, probably through complex pain pathways. It seems hard to formulize these relationships until elucidation of the precise peripheral and central neurobiological signatures in FM and other pain conditions.

## Data Availability

The datasets generated and/or analyzed during the current study are not publicly available due **to confidentiality issues** but are available from the corresponding author on reasonable request.
